# A case of burn evisceration with full-thickness injury to abdominal wall, bowel, bladder, and three extremities

**DOI:** 10.1186/s40792-021-01302-8

**Published:** 2021-09-29

**Authors:** Davit Shahmanyan, Matthew T. Joy, Bryan R. Collier, Emily R. Faulks, Mark E. Hamill

**Affiliations:** 1 Department of Surgery, Virginia Tech Carilion School of Medicine, 3 Riverside Circle, Roanoke, VA 24016 USA; 2 Present Address: Department of Surgery, University of Nebraska Medical Center, 983280 Nebraska Medical Center, MSB 4553, Omaha, NE 68198-3280 USA; 3 Department of Surgery, University of California San Francisco - Fresno, 155 N Fresno Street, Fresno, CA 93701 USA

**Keywords:** Abdominal burn, Electric injury, Bladder injury, Exploratory laparotomy abdominal wall defect, Visceral injuries, Electrical shock

## Abstract

**Background:**

Severe electrical burns are a rare cause of admission to major burn centers. Incidence of electrical injury causing full-thickness injury to viscera is an increasingly scarce, but severe presentation requiring rapid intervention. We report one of few cases of a patient with full-thickness electrical injury to the abdominal wall, bowel, and bladder.

**Case report:**

The patient, a 22-year-old male, was transferred to our institution from his local hospital after sustaining a suspected electrical burn. On arrival the patient was noted to have severe burn injuries to the lower abdominal wall with evisceration of multiple loops of burned small bowel as well as burns to the groin, left upper, and bilateral lower extremities. In the trauma bay, primary and secondary surveys were completed, and the patient was taken for CT imaging and then emergently to the operating room. On exploration, the patient had massive full-thickness burns to the lower abdominal wall, five full-thickness burns to small bowel, and intraperitoneal bladder rupture secondary to full-thickness burn. The patient underwent damage-control laparotomy including enterectomies, debridement of bladder coagulative necrosis, and layered closure of bladder injury followed by temporary abdominal closure with vacuum dressing. The patient also underwent right leg escharotomy and partial right foot fasciotomies. The patient was subsequently transferred to the nearest burn center for continued resuscitation and comprehensive burn care.

**Conclusion:**

Severe electrical burns can be associated with devastating visceral injuries in rare cases. Though uncommon, these injuries are associated with very high mortality rates. The authors assert that rapid evaluation and initial stabilization following ATLS guidelines, damage-control laparotomy, and goal-directed resuscitation in concert with transfer to a major burn center are essential in effecting a successful outcome in these challenging cases.

## Background

Severe electrical burns are rare, accounting for just 5% of admissions to major burn centers [1]. Visceral injuries related to electrical burns are even more rare with reported rates as low as 0.4% [2]. These injuries are generally associated with direct contact to high-voltage currents, most commonly in industrial accidents. Visceral injuries can occur with or without evisceration due to conduction of electrical current through the body. Electric current causes tissue damage by heat-induced coagulative necrosis, electroporation of cell membranes, and electroconformational denaturation of proteins. Mortality rates are high and correlate with the severity of injury. We present a case of a major electrical injury with abdominal wall, visceral, bladder and multiple extremity involvement after a motor vehicle crash.

## Case report

The patient was a 22-year-old male who presented to a rural hospital with a presumed high-voltage electrical injury of unclear etiology with an obvious evisceration. By report he was found walking in a dazed state unable to recount the mechanism for his injuries. He was transported via EMS to the local hospital where he was intubated and volume resuscitation was initiated. Based on the severity of his abdominal injuries, he was transported to the nearest regional level 1 trauma center for further management. Aeromedical transport was not available due to weather factors resulting in an approximately 105 min ground transport.

**Fig. 1:**
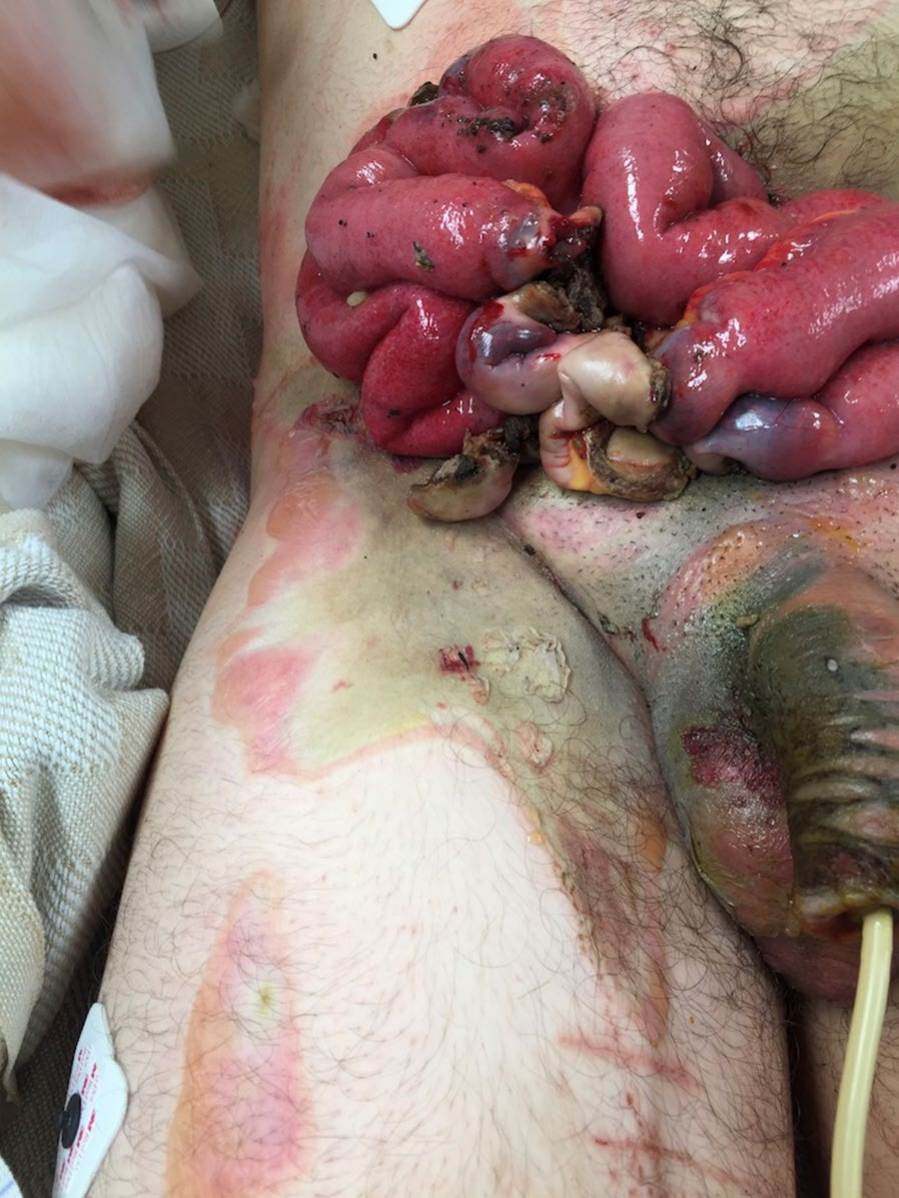
Full-thickness abdominal wall electrical burn with evisceration and bowel injury

**Fig. 2:**
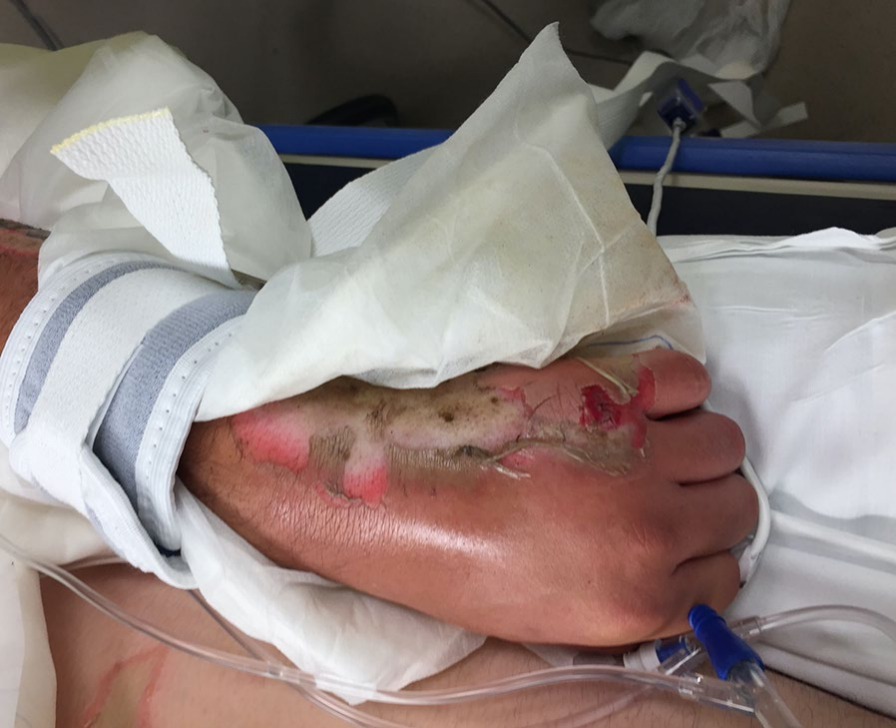
Electrical injury to left hand and forearm

**Fig. 3:**
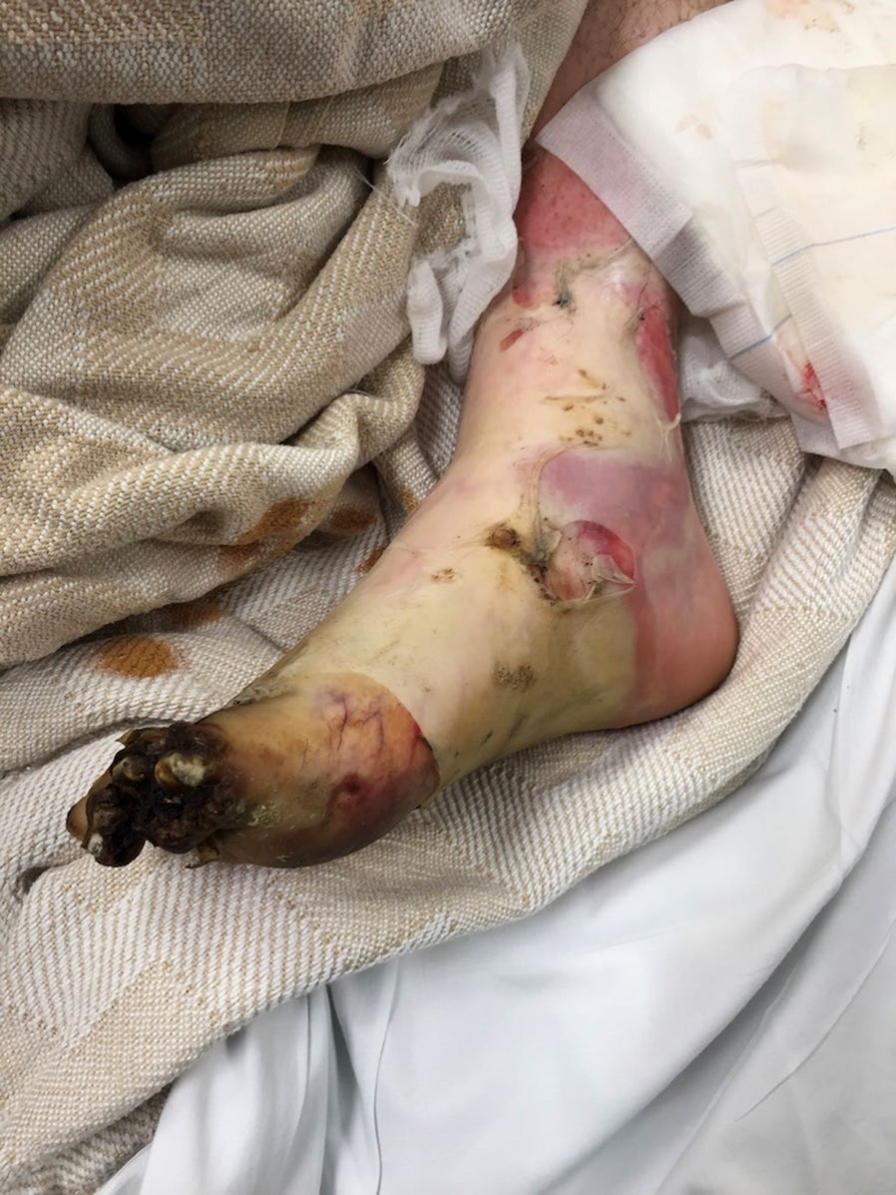
Right lower extremity full-thickness electrical burn with distal necrosis

**Fig. 4:**
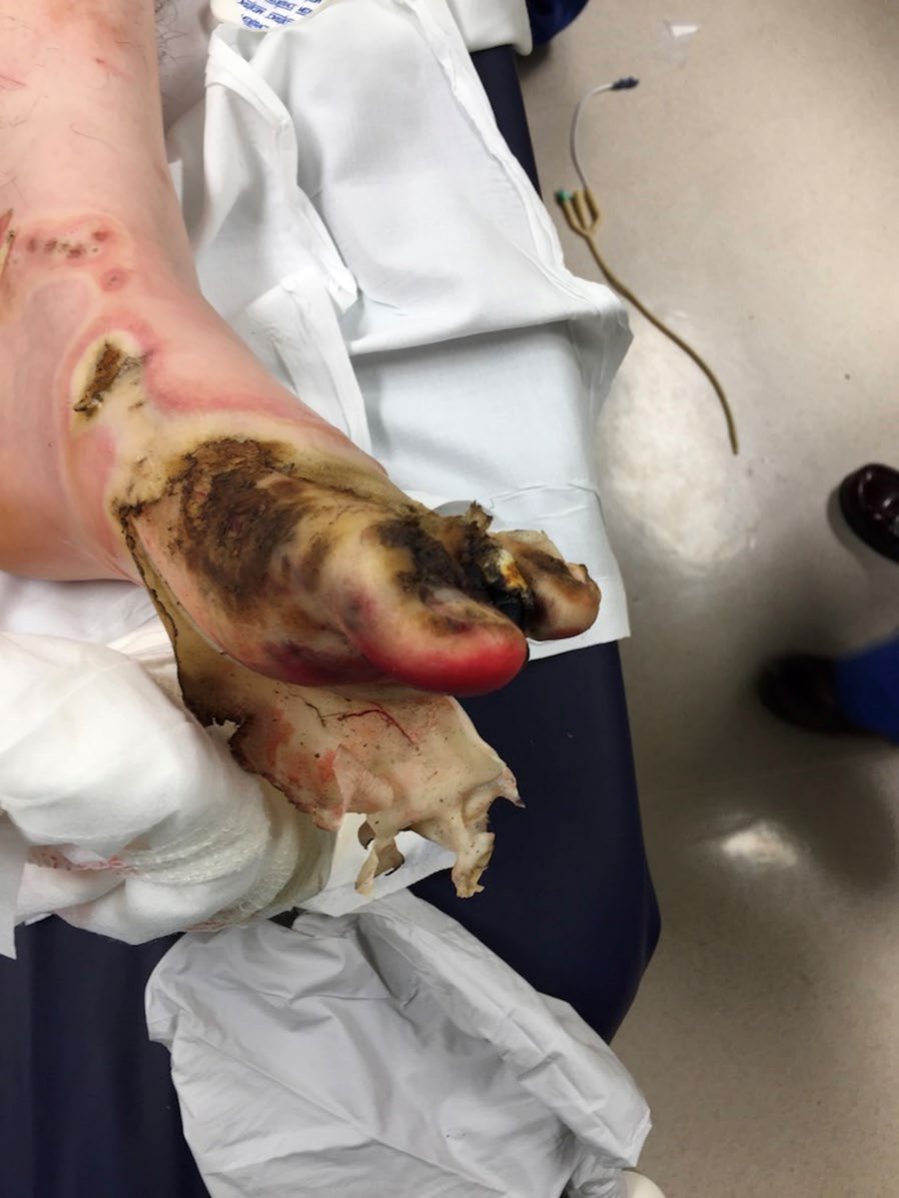
Left lower extremity full-thickness electrical burn with distal necrosis

On arrival to our facility he was hemodynamically stable, and a primary survey did not reveal any immediately life-threatening physiologic abnormalities. He did have evidence of major abdominal and perineal injuries with evisceration (Fig. 1) as well as apparent electrical injuries to his left upper (Fig. 2) and bilateral lower extremities (Figs. 3, 4). Given his unclear mechanism, CT imaging was performed to exclude other occult injuries. Imaging excluded major head, thoracic, spinal column or vascular injuries. He was then taken emergently to the operating room for abdominal exploration and other indicated procedures.

**Fig. 5:**
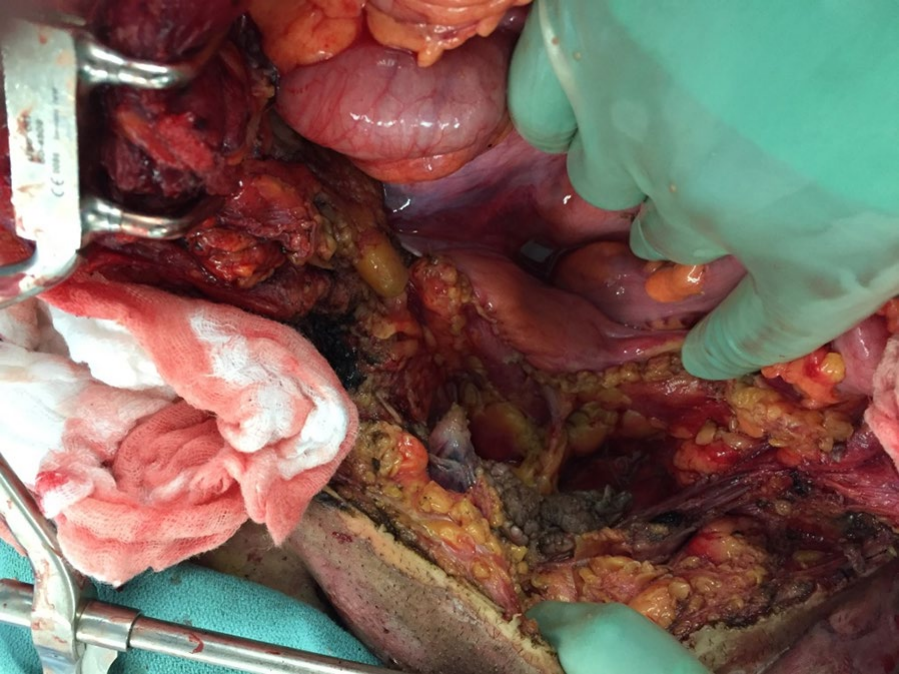
Necrosis of rectus muscle secondary to electrical injury

**Fig. 6:**
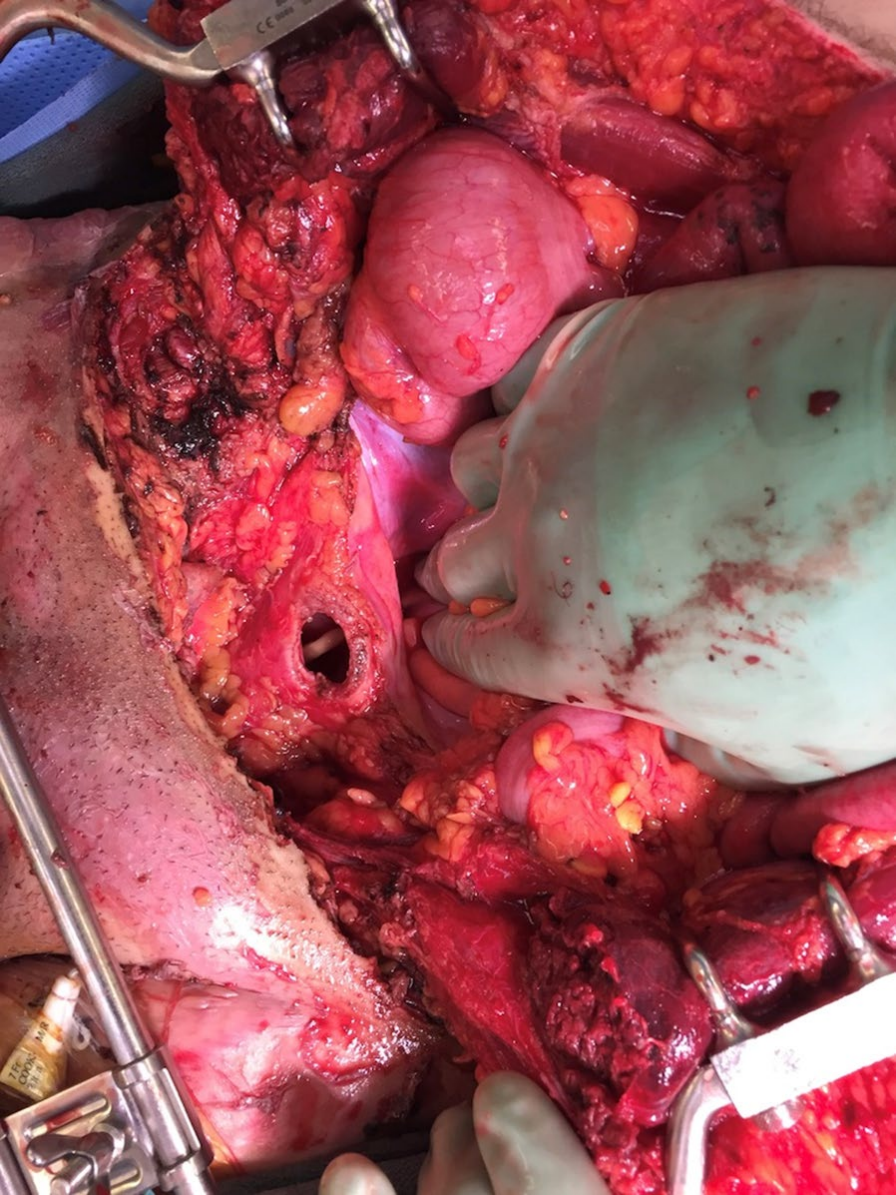
Intraperitoneal bladder perforation secondary to electrical burn

**Fig. 7:**
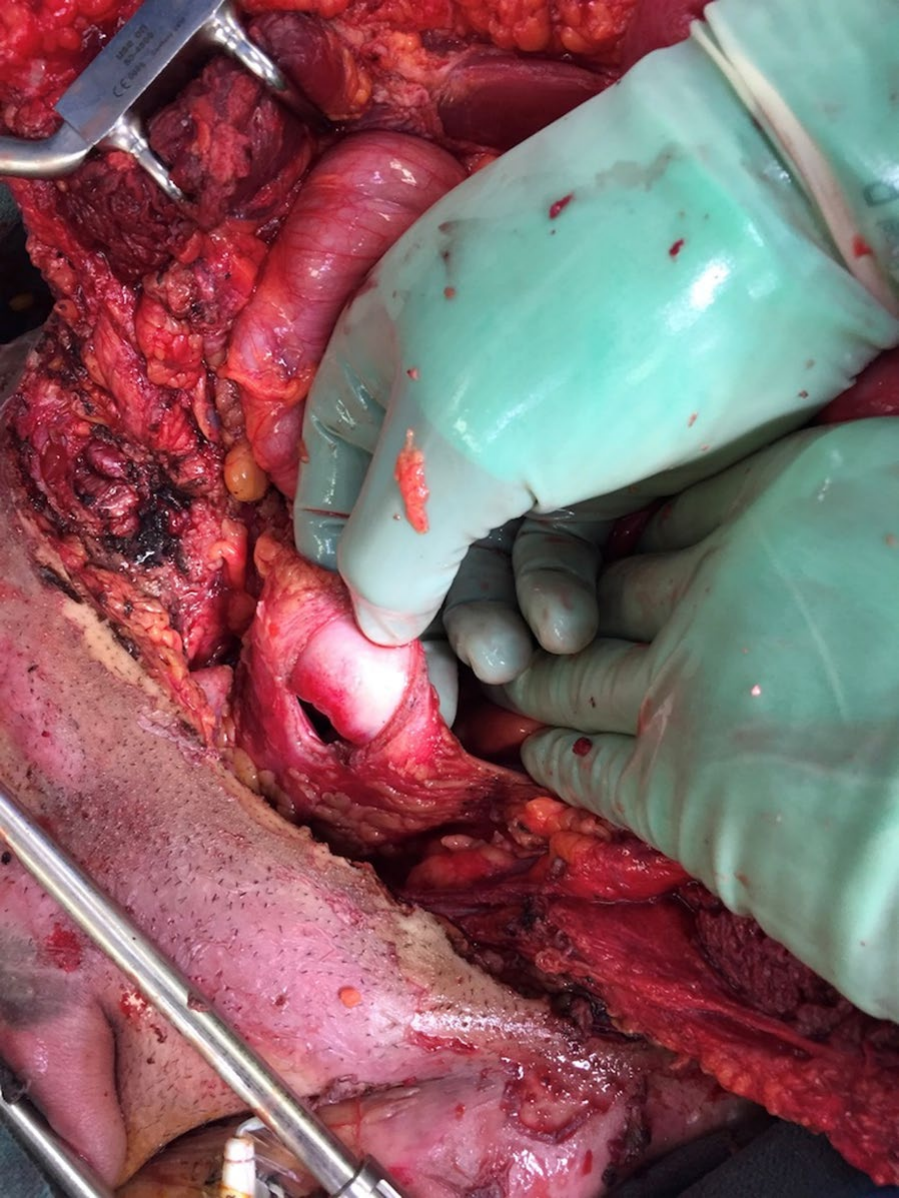
Coagulative necrosis of bladder wall secondary to electrical burn

In the operating room, a midline incision was extended from the superior aspect of the evisceration wound to the xiphoid process. Abdominal exploration revealed full-thickness burns to the abdominal wall musculature with destruction of all muscular layers of the lower abdominal wall including the rectus abdominus, the posterior rectus sheath as well as portions of the internal and external obliques (Fig. 5). While running the small bowel, five discrete areas of full-thickness coagulative necrosis were encountered. Examination of the pelvis revealed an intraperitoneal bladder injury to the dome of the bladder (Fig. 6) with obvious coagulative necrosis to the area immediately surrounding the intraperitoneal injury (Fig. 7). Multiple small bowel resections were performed, and the bowel was left in discontinuity. No attempt was made to reestablish bowel continuity due to the potential for extension of the areas of bowel necrosis and a desire to maximize length of small bowel that remained. Intraoperative consultation with urology was obtained and obvious areas of bladder necrosis were debrided. The bladder dome was closed in multiple layers with chromic sutures. A Foley catheter was left in place for bladder drainage after the repair was completed. No evidence of other hollow viscus injury, significant solid organ injury or retroperitoneal hematoma was observed. A temporary abdominal closure was then performed using a negative pressure abdominal closure system.

Following the temporary abdominal closure, our attention was then turned to the burned extremities. The patient appeared to have full-thickness circumferential injuries to the left upper extremity, left lower extremity, right lower extremity, and right foot with significantly decreased peripheral pulse exams. Intraoperative orthopedic and podiatry consults were obtained. Given the concern for compartment syndrome and potential constriction from the burned tissue, left upper extremity and bilateral lower extremity escharotomies were performed as well as right foot fasciotomies. Dry sterile dressings were applied to all wounds at the completion of the procedure. In its entirety, the initial operative procedure took 125 min. Following the procedure the patient was left intubated and arrangements were then made to transfer the patient to a regional burn center for further management. After a brief delay in setting up aeromedical transport, the patient was transferred via helicopter for further management with a 45-min transport time.

During his subsequent 2-month burn center admission, the patient required additional small bowel resections, but eventually had restoration of bowel continuity. The urinary tract injuries required vesicostomy, ureteral stenting, and revision of the original bladder repair. His clinical course was also complicated by vesiculocutaneous fistula and progression of his lower extremity injuries, eventually requiring right below knee and left transmetatarsal amputations. After successful abdominal wall reconstruction, the patient ultimately survived his injuries and was discharged from the burn center to a rehabilitation facility. More detailed information regarding his burn center hospital course is not available due to privacy issues raised by the burn center. He has subsequently been lost to follow-up at our facility.

Follow-up regarding the injury mechanism revealed that the patient was the driver of a motor vehicle which struck a power pole resulting in a downed high-voltage power line over his vehicle. By report he was initially uninjured, however when he attempted to exit his vehicle, he contacted the electrified auto frame resulting in his electrical injuries. He initially walked from the scene to a local house where EMS was contacted.

## Discussion

Abdominal burns are a rare but severe manifestation from high-voltage electrical contact burn injuries due its greater cross-sectional area and low electrical resistance [3]. The time to rapid assessment and control of abdominal wall and visceral injuries by operative debridement and resection of burned viscera and tissues was essential to the positive outcome in this case. The difficulty of assessing the depth and breadth of involvement of burned tissues has been shown to necessitate a low threshold for surgical exploration [3].

As is described in the case, subsequent to the patient’s transfer to our regional burn center it was learned that the patient’s mechanism involved a motor vehicle crash with a power pole resulting in a power line contacting the vehicle. In the United States, residential medium voltage distribution power lines typically provide between 7500 and 13,800 V before being stepped down to 120 or 240 V at the house. High-voltage power transmission lines can exceed this by a wide range—between 23,000 and 765,000 V. Alternating current at 60 Hz is supplied from the generation facility, with the occasional applications which require direct current being converted from alternating to direct current near the point of use. In this case, it is unclear exactly what voltage was involved; however, it is clear from the magnitude of the resulting injuries that high voltage was involved.

In cases without obvious visceral lesion, surgical exploration is necessitated due to the segmental and jumping nature of electrical burns [4]. The high-voltage electrical current is believed to cause coagulative necrosis in tissues due to generation of heat. Large vessels, such as the aorta, are typically spared due to low resistance resulting in minimal damage [5]. In contrast, small vessels cause high resistance explaining a higher generation of heat from electric current causing coagulative necrosis as seen in the bowel and bladder in this case. In this case, it is not possible to determine exact point of contact to the right upper extremity or abdominal wall given the degree of tissue damage which occurred to all areas. Regardless, it is clear that the electrical current caused multiple devastating injuries.

Exploratory laparotomy should not be delayed for fears of the burn injuries causing contamination of the abdominal cavity. Early surgical exploration has been shown to be essential to uncovering necrotic or nonviable tissue which acts as niduses for intra-abdominal infection [6]. If exploration is delayed, abdominal compartment syndrome may occur [7]. Subsequent amputation of necrotic extremities should be performed once resuscitation is appropriately completed [6]. In this particular case, the need for abdominal exploration was obvious given his evisceration and obvious full-thickness coagulative bowel injury. However, it is also important to remember that intra-abdominal injury resulting in the need for surgical exploration—specifically occult visceral injury or abdominal compartment syndrome—can occur in the absence of severe abdominal wall injury [1]. Radiographic findings which might indicate the need for abdominal exploration can include free air, free fluid not explained by solid organ injury, evidence of major solid organ injury, major vascular injury and mesenteric injury. Surgical procedures to extremities may be indicated on suspicion of compartment syndrome requiring fasciotomy or vascular compromise by burned tissue requiring escharotomy. In this case, both fasciotomy and escharotomy were performed in an attempt at limb salvage.

There are three described cases of bladder injury secondary to electrical injury [8–10]. Bajaj et al. describe an anterior bladder wall injury causing posterior bladder wall herniation through the abdominal wall [8]. The presentation was delayed 3 months after initial discharge for electrical injury and was treated with bladder repositioning and single-layer reconstruction with advancement of a tensor fascia lata flap. The only other described case in the literature is an enterocutaneous fistula with large interconnecting pelvic abscess noted 6 days post-electrical injury [9]. This patient was treated successfully with a 75-cm resection of ileum with end-to-end anastomosis, resection of enterovesical fistula from the dome of the bladder with primary closure and establishment of a suprapubic cystostomy. The only sign of urinary tract injury in these two cases were hematuria and myoglobinuria within the first few days of admission. The only prior characterization of acute bladder injury secondary to electrical injury was completed by Hu et al. in an 11-year-old male [10]. The authors describe electrical injury to the lower abdomen that resulted in a 3-cm longitudinal laceration of the anterior bladder wall. The bladder wound was closed primarily and successfully covered with a combined right tensor facia lata and ilioinguinal flap. It is unclear currently which treatment modality for bladder repair is optimal.

Regarding the reconstruction of the abdominal wall, prior case reports and series have demonstrated multiple strategies. These have included allowing to heal by secondary intention, acellular dermal matrix, myocutaneous rotational flaps, as well as ilioinguinal flap and tensor fascia lata muscle flaps [6, 10–12]. Given the limited patient information provided by our regional burn center, it is unclear exactly how our patient had abdominal wall reconstruction performed. Given the complexity and variability of electrical abdominal wall injury, it is doubtful that a general optimal strategy can be defined. Rather, it is likely that the strategy needs to be individualized based on the specifics of individual injuries and the local expertise available.

The medical decision-making reflected in this case is concordant with the guidelines provided within a recent review on management of abdominal electrocution [1]. Given the clear evidence of bowel injury, ATLS principles were utilized and ultimately resulted in timely damage-control laparotomy and goal-directed resuscitation. This likely helped lead to patient survival in our case.

## Conclusions

Severe electrical burns can be associated with visceral injuries in rare cases. These injuries are associated with very high rates of morbidity and mortality [1]. The authors assert that rapid evaluation and stabilization following Advanced Trauma Life Support guidelines, damage-control laparotomy to control potential sources of sepsis, and expedited transfer to a multidisciplinary burn center are essential in effecting successful outcomes in these challenging cases.

## Data Availability

All data generated or analyzed during this study are included in this published article.

## Abbreviations

ATLS: Advanced trauma life support
EMS: Emergency medical services
CT: Computed tomography

## References

[1] Marques EG, Júnior GAP, Neto BFM, Freitas RA, Yaegashi LB, Almeida CEF (2014). Visceral injury in electrical shock trauma: proposed guideline for the management of abdominal electrocution and literature review. Int J Burns Trauma..

[2] Haberal M, Uçar N, Bayraktar U, Oner Z, Bilgin N (1996). Visceral injuries, wound infection and sepsis following electrical injuries. Burns.

[3] Srivastava RK, Kumar R (2013). Electrical burns of the abdomen. Indian J Plast Surg.

[4] Zhang W, Xie W, Min W, Wang D, Zhang J, Wan S (2013). Treatment of thoracic and abdominal cavity perforation complicated by Henoch-Schonlein purpura nephritis in a patient with high-voltage electric burn. Chinese J Burn.

[5] Kumar S, Thomas S, Lehri S (1993). Abdominal wall and stomach perforation following accidental electrocution with high tension wire: a unique case. J Emerg Med.

[6] Agrawal V, Jha A, Kumar K, Kalra G (2015). Abdominal wall blow out causing bowel evisceration due to high voltage electrocution: a unique presentation. Indian J Burn.

[7] Geary SP, Spencer T, Tilney PVRR (2015). A 26-year-old man struck by lightning. Air Med J.

[8] Bajaj SP, Pande S, Ahmad I, Arora S (2000). Abdominoperineal electrical injury involving urogenital organs. Burns.

[9] Miller FE, Peterson D, Miller J (1986). Abdominal visceral perforation secondary to electrical injury: case report and review of the literature. Burns.

[10] Hu D, Meng C, Hu J, Zhou Y, Lu S, Yu Y (2019). Ilioinguinal flap combined with tensor fascia lata muscle flap to repair deep electrical burns in the lower abdomen: a report of two cases. Wounds.

[11] Xiao SC, Zhu SH, Li HY, Wang GY, Xia ZF (2009). Repair of complex abdominal wall defects from high-voltage electric injury with two layers of acellular dermal matrix: a case report. J Burn Care Res.

[12] Zhang PH, Liu Z, Ren LC, Zeng JZ, Huang GW, Xiao MZ, Zhou J, Liang PF, Zhang MH, Huang XY (2017). Early laparotomy and timely reconstruction for patients with abdominal electrical injury: five case reports and literature review. Medicine (Baltimore).

